# DNA combing versus DNA spreading and the separation of sister chromatids

**DOI:** 10.1083/jcb.202305082

**Published:** 2024-02-05

**Authors:** Alice Meroni, Sophie E. Wells, Carmen Fonseca, Arnab Ray Chaudhuri, Keith W. Caldecott, Alessandro Vindigni

**Affiliations:** 1 Division of Oncology, Department of Medicine, Washington University in St. Louis School of Medicine, St. Louis, MO, USA; 2 Genome Damage and Stability Centre, School of Life Sciences, University of Sussex, Falmer Brighton, UK; 3 Department of Molecular Genetics, Erasmus MC Cancer Institute, Erasmus University Medical Center, Rotterdam, Netherlands

## Abstract

DNA combing and DNA spreading are two central approaches for studying DNA replication fork dynamics genome-wide at single-molecule resolution by distributing labeled genomic DNA on coverslips or slides for immunodetection. Perturbations in DNA replication fork dynamics can differentially affect either leading or lagging strand synthesis, for example, in instances where replication is blocked by a lesion or obstacle on only one of the two strands. Thus, we sought to investigate whether the DNA combing and/or spreading approaches are suitable for resolving adjacent sister chromatids during DNA replication, thereby enabling the detection of DNA replication dynamics within individual nascent strands. To this end, we developed a thymidine labeling scheme that discriminates between these two possibilities. Our data suggests that DNA combing resolves sister chromatids, allowing the detection of strand-specific alterations, whereas DNA spreading typically does not. These findings have important implications when interpreting DNA replication dynamics from data obtained by these two commonly used techniques.

## Teaser

DNA combing and DNA spreading are two central approaches for studying DNA replication fork dynamics genome-wide at single-molecule resolution. Meroni et al. show that DNA combing resolves sister chromatids, allowing the detection of strand-specific alterations, whereas DNA spreading typically does not.

## Introduction

DNA replication is the key process that ensures cell division and the correct propagation of genetic information to daughter cells. This process entails the transient unwinding of the DNA duplex, leading to the formation of a three-way junction structure within which the separated parental DNA strands serve as a template for the synthesis of the newly formed leading and lagging strand fragments. This process is mediated by a complex of proteins termed the “replisome” (Yao and O’Donnell, 2009).

One of the most powerful and widely used techniques to monitor DNA replication fork dynamics at single-molecule resolution is the DNA fiber spreading assay, which relies on the ability of replicating cells to incorporate thymidine analogs in the newly synthesized (nascent) strands. Briefly, nascent strands are sequentially pulse-labeled with two labeled thymidine analogs, typically chosen from 5-iodo-2-deoxyuridine (IdU), 5-chloro-2′-deoxyuridine (CldU), or 5-bromo-2-deoxyuridine (BrdU). Cells are then lysed to release and deposit their DNA on the positively charged surface of the glass slide. The slide is then tilted at a 25–60° angle to favor the spreading of the DNA by gravity (Parra and Windle, 1993). Alternatively, in a variation of DNA spreading known as DNA combing, the labeled cells are embedded in agarose plugs from which DNA is extracted, usually with the aid of proteinase K treatment, and “combed” in uniform parallel arrays with the aid of a combing machine on a silanized coverslip (Bensimon et al., 1994).

After spreading or combing, the labeled DNA is visualized through immunofluorescence by incubation with antibodies that specifically recognize the halogenated nucleosides incorporated in the DNA. The length of individual DNA fibers can be measured at a single molecule level as a direct readout of DNA replication fork progression (Técher et al., 2013). A fiber of ∼1 µm corresponds to 2.6 kb of DNA with the DNA spreading technique (Daigaku et al., 2010; Jackson and Pombo, 1998) and ∼2 kb for DNA combing (Bensimon et al., 1994; Michalet et al., 1997). In addition to DNA replication fork progression, other replication parameters can be evaluated using the DNA fiber approach including fork symmetry, origin firing, and nascent DNA degradation (reviewed in Quinet et al. [2017]).

DNA spreading is less time-consuming than DNA combing and is generally regarded as a higher-throughput technique. On the other hand, DNA spreading leads to a non-uniform distribution of the DNA on the slides, whereas DNA combing leads to a parallel and uniform distribution of DNA molecules. This uniform distribution enables more robust and accurate measurements of the length of the DNA fibers, which is important when measuring parameters such as the DNA replication velocity or the distance between two replication origins within the same filament (Técher et al., 2013).

**Figure 1.**
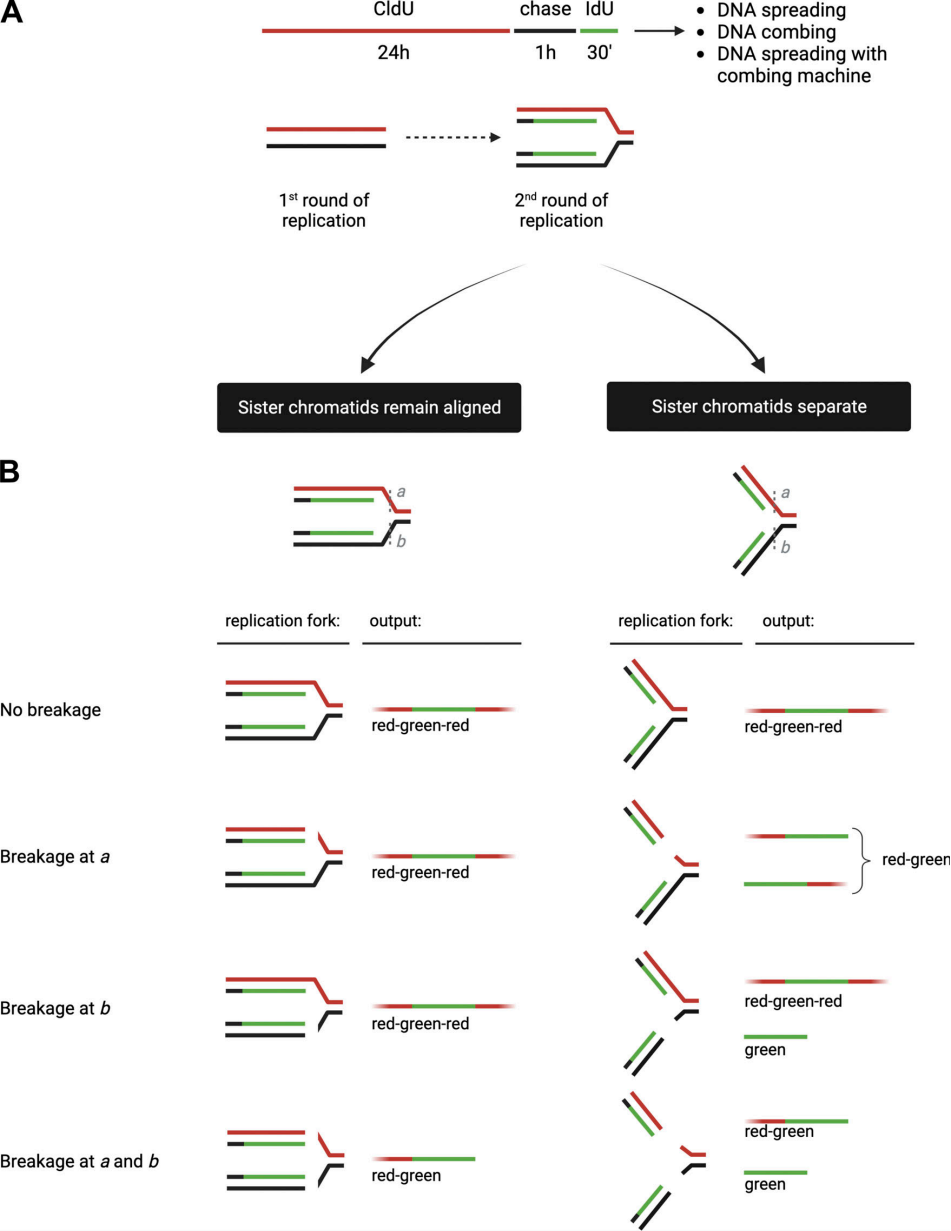
**Schematic and model for defining the outcome of sister chromatid alignment versus separation. (A)** Use of halogenated thymidine analogs to distinguish between sister chromatids during S phase. RPE-1 and U2OS cells are incubated with 1 µM CldU for 24 h (first round of replication), chased with fresh media for 1 h, and incubated with 200 µM IdU for 30 min (second round of replication). **(B)** Predicted outcomes for sister chromatid alignment (left) versus separation (right). If sister chromatids remain aligned/adjacent to each other (left), they will result in DNA fibers that are red–green–red if there is no breakage, breakage at a, or breakage at b, and in fibers that are red–green upon breakage at both at a and b. In contrast, if the sister chromatids become separated (right), they will result in DNA fibers that are red–green–red if there is no breakage, red–green upon breakage at a, red–green–red and green only upon breakage at b, and red–green and green only upon breakage at both a and b.

An important consideration is that the thymidine analogs are simultaneously incorporated on both the leading and lagging strands. However, the genome is constantly under the attack of endogenous and exogenous agents that can lead to DNA damage and perturb DNA replication dynamics (Berti et al., 2020; Cybulla and Vindigni, 2023). These lesions or replication obstacles can be located in one or both template DNA strands and thereby differentially affect either the leading or the lagging strand synthesis. Here, we combined the efforts of three independent laboratories to evaluate whether DNA spreading and DNA combing assays can resolve distinct DNA sister chromatids. This is important because DNA replication defects in only one of the two sister chromatids, such as a broken sister chromatid arising from DNA replication fork collapse at a single-strand break in one of the two DNA template strands, can only be detected if the two adjacent sister chromatids are separated during the DNA fiber experiment (Fig. 1).

## Results

To determine the ability of the spreading and combing assays to separate the two sister chromatids, we designed a labeling scheme in which we first incubated human retinal pigment epithelial cells (RPE-1) cells with CldU (red label) for 24 h to allow a single round of DNA replication and thus label one DNA strand of the entire genome of all cells with CldU. Next, we incubated the cells for 30 min with IdU (green label) to pulse-label active DNA replication forks during the second round of DNA replication (Fig. 1 A). Importantly, we chased cells with media lacking labeled nucleoside for 1 h between the two analogs to separate CldU from IdU incorporation during the second round of replication. We then collected the cells and processed them in parallel with the DNA spreading and combing approaches. If sister chromatids are separated, we should be able to detect green-only tracts (Fig. 1 B). This is because if sister chromatids are separated, any fork breakage arising in the cells prior to harvest or during the combing/spreading procedure will result in linear fibers comprised of individual sister chromatids that allow “green-only” replication tracks to be detected (green-only tracts in Fig. 1 B). Specifically, we envision three possible scenarios with respect to such breakage: (1) absence of breakage; (2) breakage of one sister chromatid (either at position “a” or “b”); and (3) simultaneous breakage of both sister chromatids (at positions a and b) (Fig. 1 B). If sister chromatids are separated, breakage at b or at a and b will result in green-only tracts. In contrast, if adjacent sister chromatids retain their cohesion and remain closely aligned, we should only detect fibers with adjacent red and green tracks (either “red–green” or “red–green–red” tracts), irrespective of whether there is any type of fork breakage in the cell or during the combing/spreading procedure.

**Figure 2.**
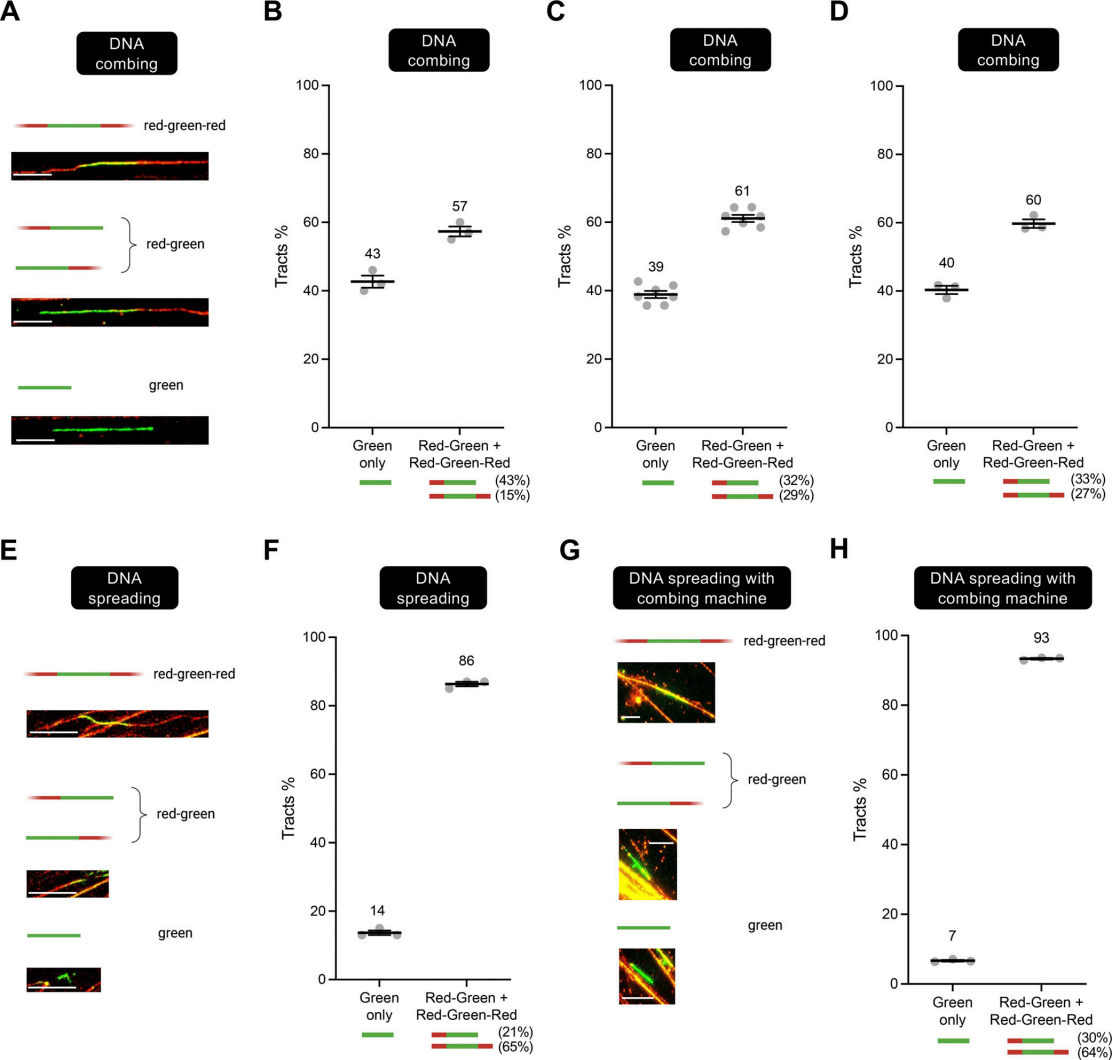
**Sister chromatids are separated during DNA combing but not spreading. (A)** Representative images of DNA fibers obtained with the combing approach. Scale bar is 10 µm. **(B–D)** DNA combing assay performed as depicted in Fig. 1 A. Green-only versus red–green or red–green–red tracts are scored and represented as percentage of total pulse-labeled tracts. At least 100 tracts were scored for each sample. **(B–D)** Independent data sets from the Vindigni, Caldecott, and Chaudhuri laboratories, respectively. *N* indicates the number of biological repeats (*N* = 3 in B, *N* = 6 in C, and *N* = 3 in D). Statistics: mean ± SEM. Numbers indicate the mean tracts %. **(E)** Representative images of DNA fibers obtained with the spreading approach. Scale bar is 10 µm. **(F)** DNA spreading assay performed by the Vindigni laboratory as depicted in Fig. 1 A. Green-only versus red–green or red–green–red tracts are scored and represented as percentage of total pulse-labeled tracts. At least 100 tracts were scored for each sample. Number of biological repeats *N* = 3. Statistics: mean ± SEM. Numbers indicate the mean tracts %. **(G)** Representative images of DNA fibers obtained with the hybrid approach where the DNA is spread on the coverslips using the combining machine. Scale bar is 10 µm. **(H)** DNA spreading with combing machine assay performed by the Chaudhuri laboratory as depicted in Fig. 1 A. Green-only versus red–green or red–green–red tracts are scored and represented as percentage of total pulse-labeled tracts. At least 100 tracts were scored for each sample. Number of biological repeats *N* = 3. Statistics: mean ± SEM.

**Figure S1.**
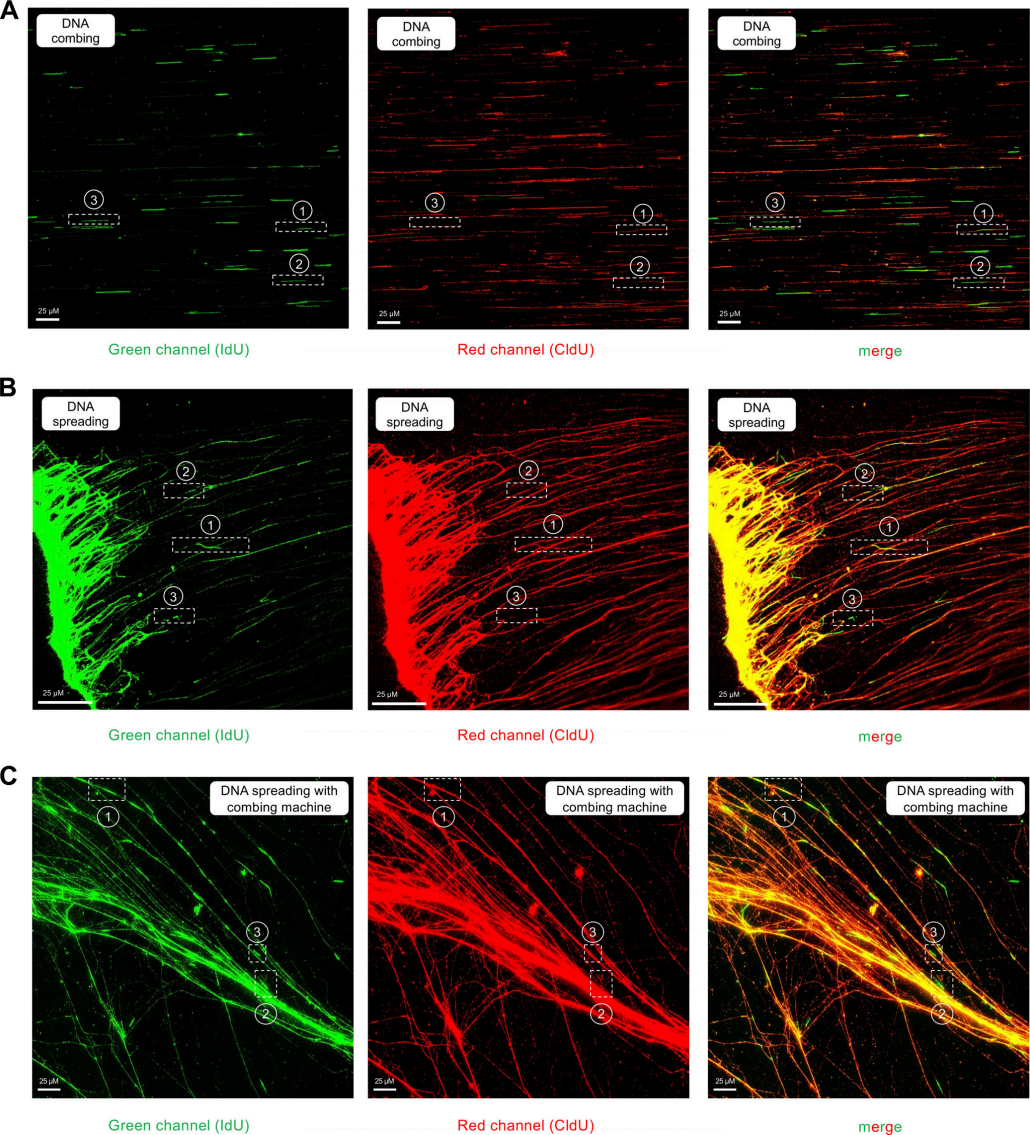
**(Related to** Fig. 2**): Examples of DNA fiber images collected with the DNA combing, spreading, and hybrid spreading techniques. (A)** Images collected with DNA combing. The numbers 1, 2, and 3 denote the examples of red–green–red, red–green, and green-only fibers shown in Fig. 2 A. **(B)** Images collected with DNA spreading. The numbers 1, 2, and 3 denote the examples of red–green–red, red–green, and green-only fibers shown in Fig. 2 E. **(C)** Images collected with the hybrid spreading technique. The numbers 1, 2, and 3 denote the examples of red–green–red, red–green, and green-only fibers shown in Fig. 2 G. In all cases, when we score red–green–red or red–green fibers, the red signal is continuous and extends through the green tracts, confirming that our labeling originates from two separate replication cycles. The reason why we do not see yellow tracts when the CldU and IdU overlap is that the IdU signal (green) is stronger than the CldU signal (red) due to the different efficiencies of the two antibodies (see also Materials and methods).

**Figure S2.**
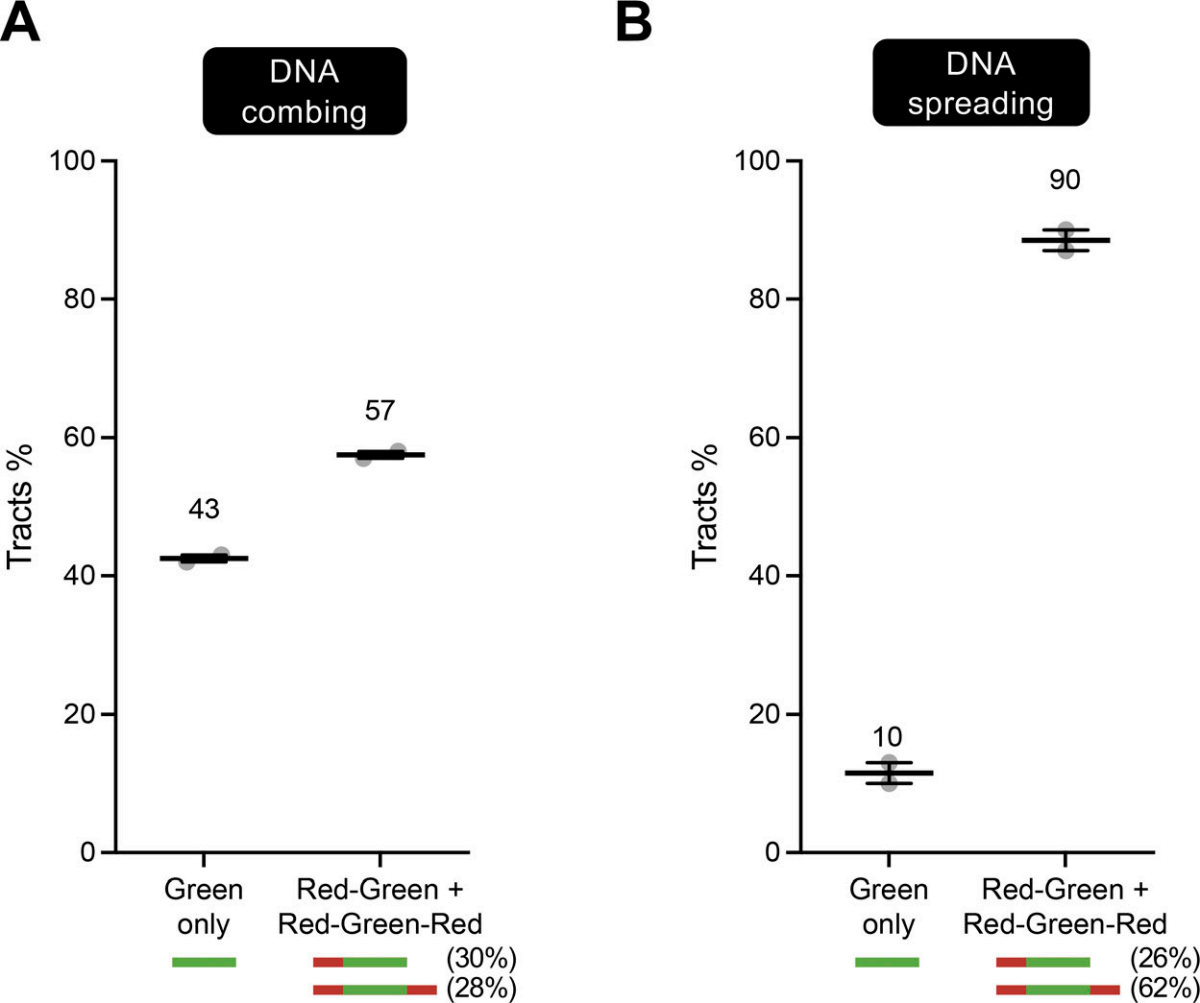
**(Related to** Fig. 2**): Sister chromatids are separated during DNA combing but not spreading in U2OS cells. (A)** DNA combing assay performed by the Vindigni laboratory as depicted in Fig. 1 A. Green-only versus red–green or red–green–red tracts are scored and represented as percentage of total pulse-labeled tracts. At least 100 tracts were scored for each sample. Numbers indicate the mean tracts %. Statistics: mean ± SEM. Number of biological repeats *N* = 3. **(B)** DNA spreading assay performed by the Vindigni laboratory as depicted in Fig. 1 A. Green-only versus red–green or red–green–red tracts are scored and represented as percentage of total pulse-labeled tracts. At least 100 tracts were scored for each sample. Numbers indicate the mean tracts %. Statistics: mean ± SEM. Number of biological repeats *N* = 3.

Consistent with our hypothesis, following DNA combing, ∼40% of labeled DNA replication tracts were green-only, with ∼60% being red–green or red–green–red (Fig. 2, A–D and Fig. S1 A). These data suggest that adjacent sister chromatids are indeed separated during DNA combing. In contrast, following DNA spreading, we observed a much lower percentage (∼14%) of green-only tracts (Fig. 2, E and F; and Fig. S1 B). Similar percentages were also obtained using human osteosarcoma U2OS cells, suggesting that the observed phenotype is not cell-type specific (Fig. S2). These data suggest that there is robust separation of homologous sister chromatids during combing, but that homologous sister chromatids largely remain adjacent during DNA spreading. Interestingly, similar results to spreading were also obtained using a hybrid protocol in which the cells were lysed on a coverslip and processed as in the spreading protocol, but the DNA from the lysed cells then spread over the coverslip using combing (Fig. 2, G and H; and Fig. S1 C). These results argue that the difference in the percentage of green-only tracts observed between combing and spreading (or hybrid spreading), and thus in the extent of sister chromatid separation, is most likely related to differences in the protocols used for sample preparation.

Notably, ∼65% of the dual-labeled tracts detected using the spreading or the hybrid approach were red–green–red, whereas only 15–29% of such tracts were red–green–red following combing. This also is consistent with the idea that sister chromatids largely remain adjacent during spreading or the hybrid approach (see Fig. 1 B), irrespective of the presence of fork breakage in one of the two DNA templates. The 21–30% of red–green tracts that we detected in our spreading and hybrid spreading experiments might reflect adjacent sister chromatids that underwent breakage on both DNA templates at a and b (Fig. 1 B).

**Figure 3.**
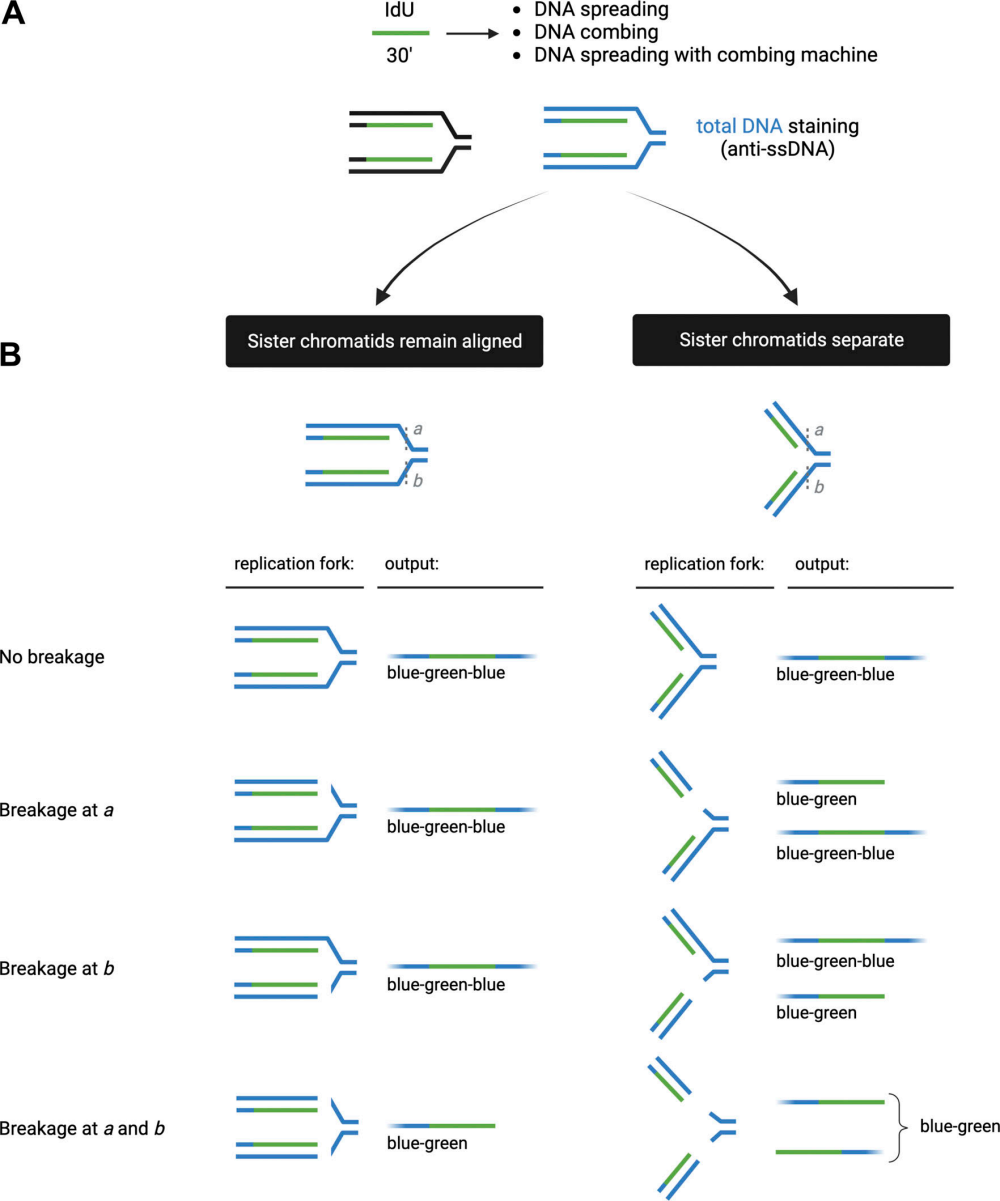
**Schematic of the total DNA staining experiment. (A)** RPE-1 cells are incubated with IdU for 30 min followed by staining with anti-IdU (green) and anti-DNA antibody (anti-ssDNA, blue) to detect the integrity of the DNA flanking the DNA replication tract. **(B)** Predicted outcomes for sister chromatid alignment (left) versus separation (right). If sister chromatids remain aligned/adjacent to each other (left), they will result in DNA fibers that are blue–green–blue if there is no breakage, breakage at a, or breakage at b, and in fibers that are blue–green upon breakage at both at a and b. In contrast, if the sister chromatids become separated (right), they will result in DNA fibers that are blue–green–blue if there is no breakage, blue–green–blue and blue–green upon breakage at a or b, and blue–green upon breakage at both a and b.

**Figure 4.**
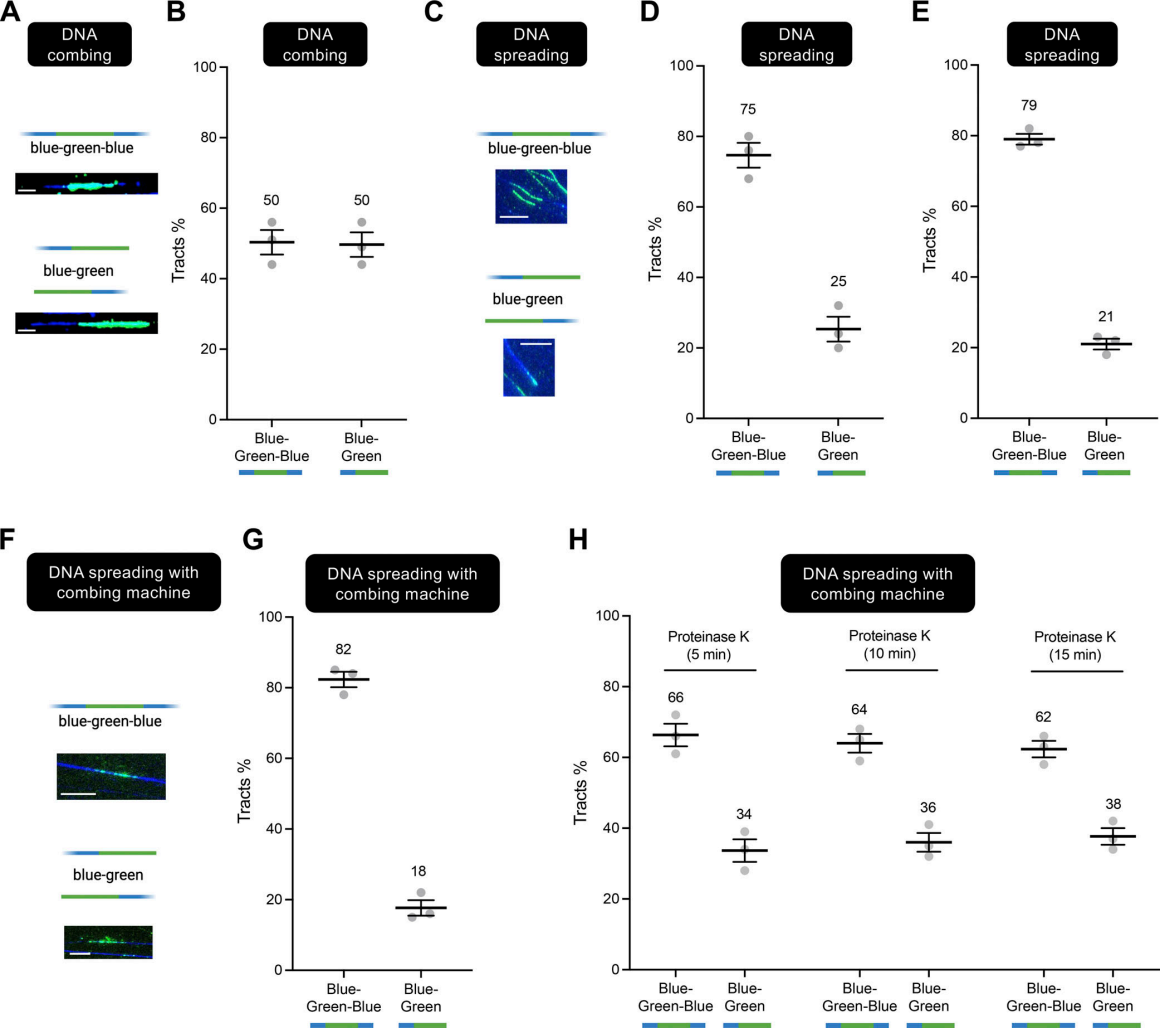
**Higher percentages of broken forks are detected by DNA combing compared to spreading, and this difference is reduced by addition of proteinase K to the spreading and hybrid spreading protocols. (A)** Representative images of DNA fibers obtained with the combing approach. Scale bar is 10 µm. **(B)** Blue–green–blue versus blue–green forks scored after combing and presented as a percentage of the total scored pulse-labeled tracts. At least 100 tracts were scored for each sample. Numbers indicate the mean tracts %. Statistics: mean ± SEM. Number of biological repeats *N* = 3. **(C)** Representative images of DNA fibers obtained with the spreading approach. Scale bar is 10 µm. **(D and E)** Blue–green–blue versus blue–green forks scored after spreading and presented as a percentage of the total scored pulse-labeled tracts. At least 100 tracts were scored for each sample. Numbers indicate the mean tracts %. D and E indicate results from the Vindigni and Chaudhuri laboratories, respectively. *N* indicates the number of biological repeats (*N* = 3 in D and *N* = 3 in E). Statistics: mean ± SEM. **(F)** Representative images of DNA fibers obtained with the hybrid spreading approach. Scale bar is 10 µm. **(G)** Blue–green–blue versus blue–green forks scored after using a hybrid spreading protocol, where the DNA is extracted following the spreading approach and combed on coverslips. Data are presented as a percentage of the total scored pulse-labeled tracts. At least 100 tracts were scored for each sample. Numbers indicate the mean tracts %. Data are from *N* = 3 independent experiments. Statistics: mean ± SEM. **(H)** Blue–green–blue versus blue–green forks scored after addition of proteinase K to hybrid spreading protocol. Data are presented as a percentage of the total scored pulse-labeled tracts. At least 100 tracts were scored for each sample. Numbers indicate the mean tracts %. Statistics: mean ± SEM. Number of biological repeats *N* = 3.

**Figure S3.**
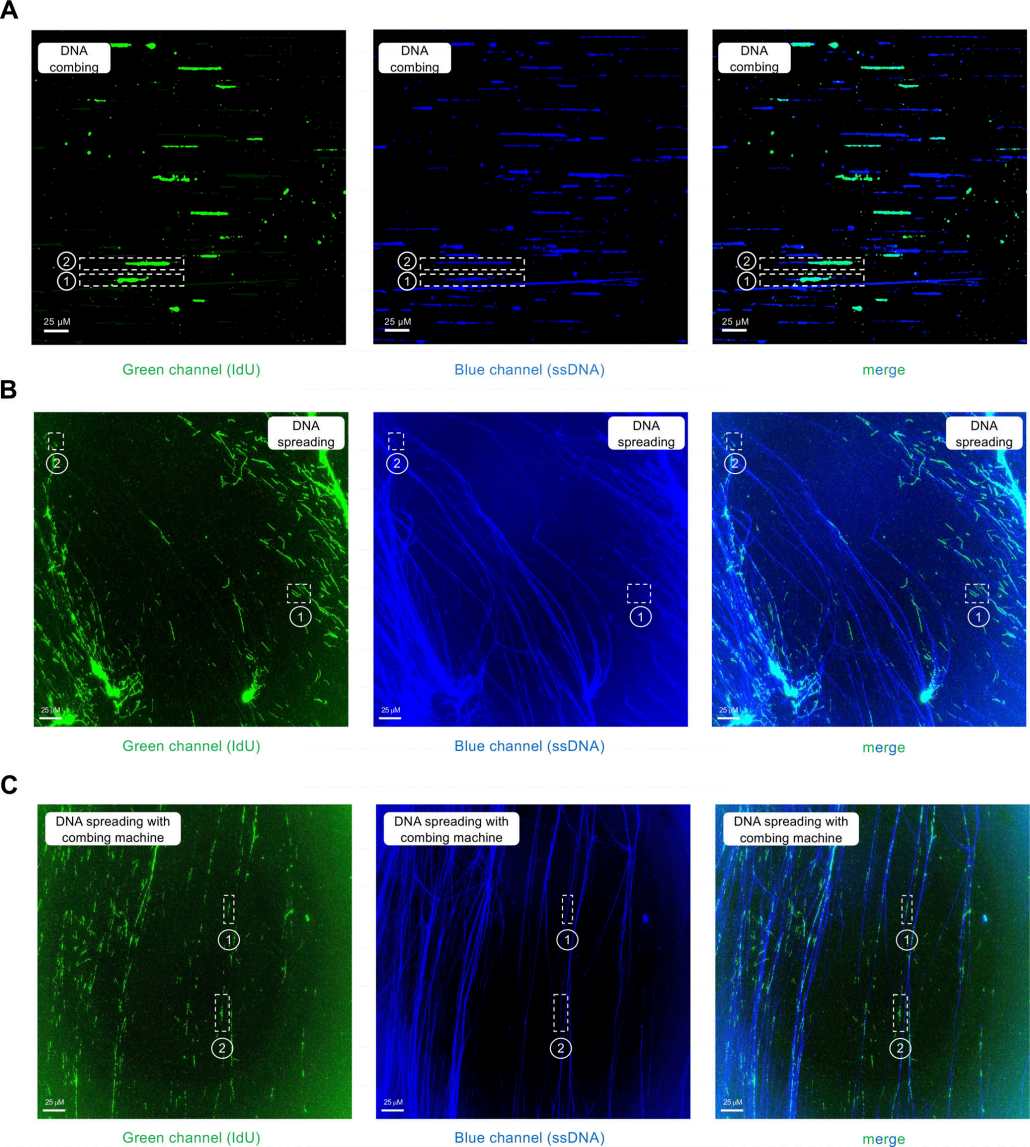
**(Related to** Fig. 4**): Examples of DNA fiber images of the total DNA staining experiments performed the DNA combing, spreading, and hybrid spreading techniques. (A)** Images collected with DNA combing. The numbers 1 and 2 denote the examples of blue–green–blue and blue–green fibers shown in Fig. 4 A. **(B)** Images collected with DNA spreading. The numbers 1 and 2 denote the examples of blue–green–blue and blue–green fibers shown in Fig. 4 A. **(C)** Images collected with the hybrid spreading technique. The numbers 1 and 2 denote the examples of blue–green–blue and blue–green fibers shown in Fig. 4 F.

The conclusions described above depend on our prediction that the appearance of green-only tracks in combing experiments reflects the breakage of one or both template strands (either before or during sample processing) at or close to the replication forks. Such breakage could reflect the known occurrence of fork breakage in cells, which is readily detected by combing (Serrano-Benitez et al., 2023) and/or breakage of forks during sample processing. To confirm that broken forks are present in DNA samples following combing, we labeled nascent DNA for 30 min with IdU followed by staining with anti-IdU (green) and anti-DNA antibody (anti-ssDNA, blue) to detect the integrity of the DNA flanking the DNA replication tract directly (Fig. 3 A). We then quantified those IdU pulse-labeled tracts that had DNA on one side only (blue–green) or on both sides (blue–green–blue). We found that, following combing, ∼50% of blue–green DNA replication tracts were blue–green and thus lacked DNA continuity on one side of the IdU (green) pulse label, consistent with fork breakage at one or both DNA template strands (Fig. 4, A and B; and Fig. S3 A).

In contrast to the combing approach, following spreading, the majority of IdU pulse labeled tracts (∼80%) were flanked by DNA (blue–green–blue), suggesting that the breakage of parental single strands at replication forks is either far less common during spreading, or perhaps more likely that fork breakage is masked by the greater retention of sister chromatid cohesion/alignment during spreading (Fig. 4, C–E; and Fig. S3 B). Consistent with the latter possibility, similar results were obtained using the hybrid protocol, which in line with DNA spreading also favors the retention of sister chromatid alignment and/or cohesion (Fig. 4, F and G; and Fig. S3 C).

With respect to how fork breakage occurs during combing/spreading experiments, we believe this reflects, at least in part, bona fide replication fork stress occurring in cells. This is because the force applied to DNA during combing/spreading is unlikely to be sufficient to break DNA molecules. For example, previous studies suggest that during combing, the receding air–water meniscus exerts a constant force on the attached DNA molecules, which is sufficient to stretch but not break DNA (Lebofsky and Bensimon, 2003; Bensimon et al., 1995; Cluzel et al., 1996; Smith et al. 1996; Strick et al., 1996). However, we cannot rule out fork breakage during sample preparation as a result of increased susceptibility of single-stranded DNA at forks to physical and/or chemical damage.

**Figure S4.**
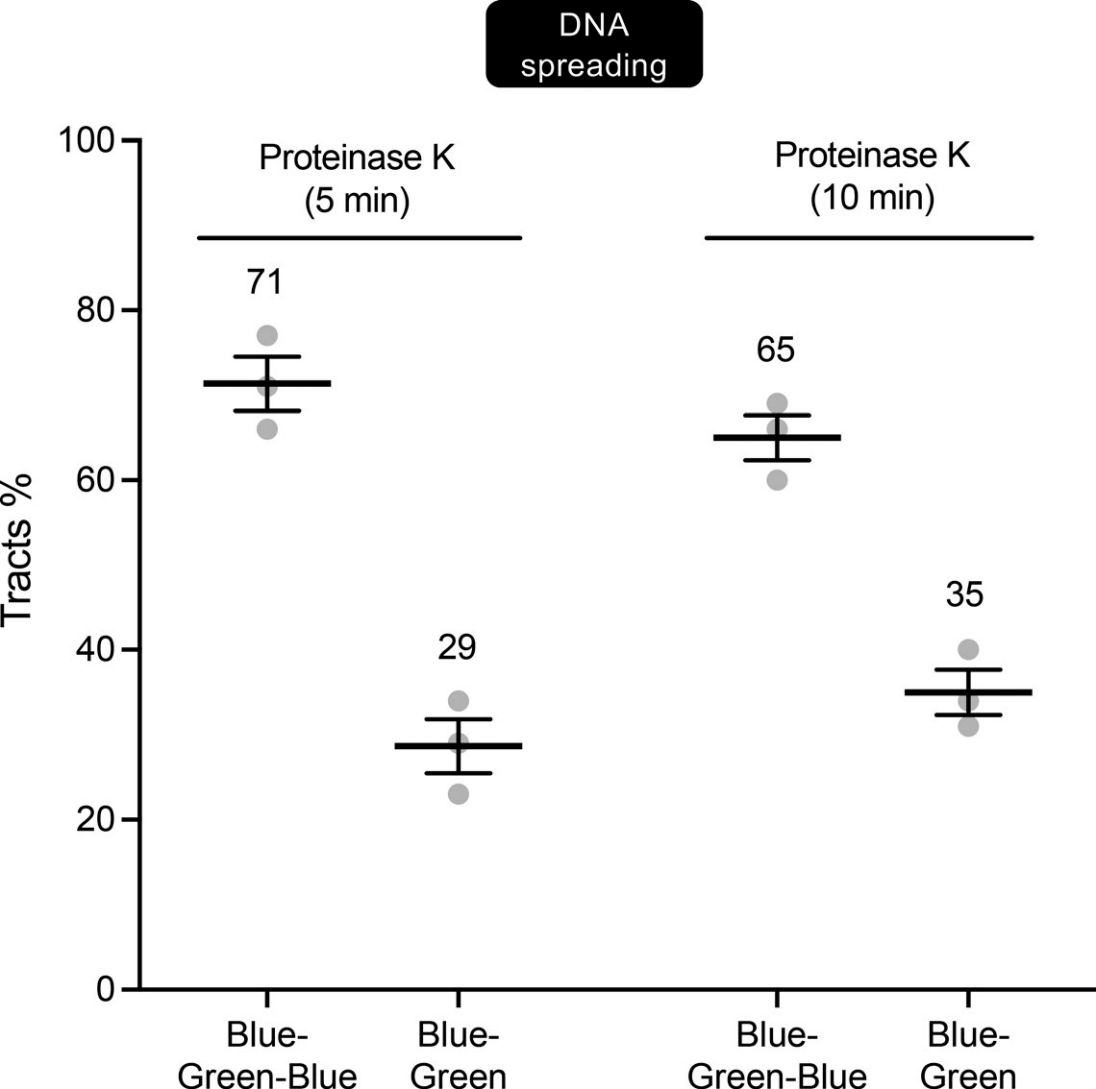
**(Related to** Fig. 4**): Addition of proteinase K increases the percentages of broken forks detected by spreading.** Blue–green–blue versus blue–green forks scored after the addition of proteinase K to the spreading protocol. DNA spreading assay performed by the Chaudhuri laboratory as depicted in Fig. 3 A. Data are presented as a percentage of the total scored pulse-labeled tracts. At least 100 tracts were scored for each sample. Numbers indicate the mean tracts %. Statistics: mean ± SEM. Number of biological repeats *N* = 3.

We reasoned that the differences observed between the combing and spreading (or hybrid) protocols may in part reflect the inclusion of overnight treatment with proteinase K during sample preparation, which likely promotes the loss of sister chromatid cohesion during the combing procedure. Consistent with this idea, the inclusion of proteinase K treatment for up to 15 min in the spreading and hybrid protocols increased the fraction of broken IdU pulse-labeled forks (blue–green) from ∼20% (without proteinase K) to ∼35% following treatment with proteinase K (Fig. 4 H and Fig. S4). Note that we could not treat samples with proteinase K for longer than 10–15 min in the spreading and hybrid protocols because longer incubation times prevented the adhesion of DNA to the slides. Collectively, these results provide further support to our model that fork breakage might not be directly responsible for the observed difference between spreading and combing, and that fork breakage might not be visible during spreading because of the continued cohesion of the sister chromatids, which can be disrupted by addition of proteinase K.

**Figure 5.**
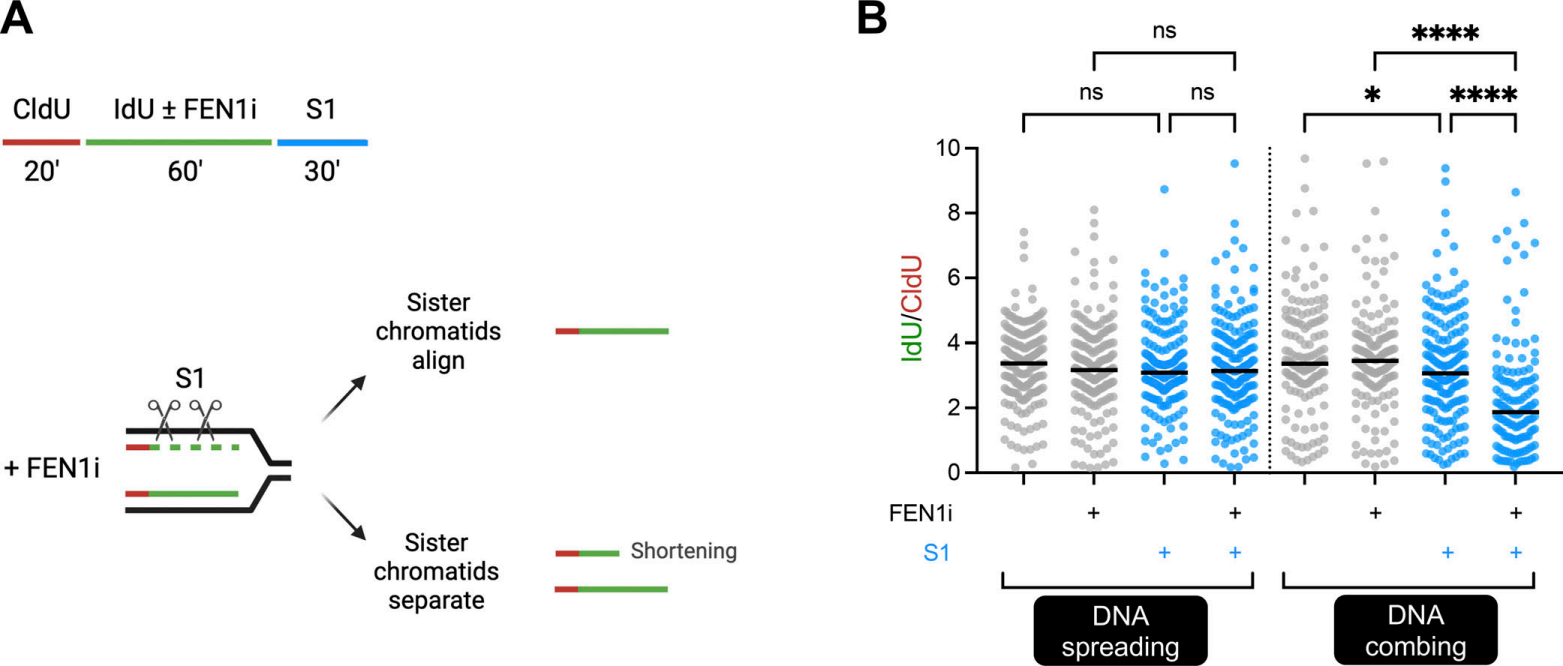
**DNA combing, but not DNA spreading, can detect DNA single-strand gaps within individual sister chromatids. (A)** Schematic depicting the use of pulse-labeling and FEN1 inhibitor (FEN1i) to detect single-strand gaps at unligated Okazaki fragments. **(B)** Dot plots of IdU/CldU ratios in cells treated ± FEN1i and ± S1 nuclease as indicated. Number of biological repeats *N* = 2. Bars represent the median values. At least 100 tracts were scored for each sample. Statistics: Kruskal–Wallis test. ns, non-significant, *P ≤ 0.05, ****P ≤ 0.0001.

As a final test of our model, we compared the ability of DNA spreading and DNA combing to detect post-replicative DNA single-strand gaps (ssDNA gaps). Post-replicative single-strand DNA gaps have emerged as a vulnerability of cancer cells and are detectable by pretreatment of genomic DNA with the single-strand specific S1 nuclease enzyme prior to DNA spreading or DNA combing (S1 fiber technique) (Quinet et al., 2016; Vaitsiankova et al., 2022). This is because the S1 nuclease cleaves the intact DNA strand at ssDNA gaps, resulting in the shortening of the DNA replication tract associated with that sister chromatid. Based on our model, S1 treatment should lead to different outcomes in DNA spreading and DNA combing experiments. Specifically, if DNA combing separates sister chromatids as we have proposed, then treatment with S1 nuclease will unveil ssDNA gaps and thus lead to detectable replication tract shortening as described above. In contrast, if DNA spreading does not separate sister chromatids, treatment with S1 nuclease will not lead to detectable shortening of DNA replication tracts because the presence of the gap will be masked by the adjacent sister chromatid, except in the situation of closely apposed ssDNA gaps in both adjacent sister chromatids. To test this hypothesis, we treated cells with an inhibitor of the Okazaki fragment processing enzyme FEN1 nuclease (FEN1i) (Exell et al., 2016; Goulian et al., 1990; Harrington and Lieber, 1994; Tumey et al., 2005), which we have shown previously can slow the maturation of Okazaki fragments resulting in ssDNA gaps on nascent lagging strands (Vaitsiankova et al., 2022; Hanzlikova et al., 2018). As predicted by our model, upon pulse labeling of RPE-1 cells with CldU for 20 min, followed by labeling with IdU for 60 min in the presence or absence of FEN1 inhibitor (FEN1i), we failed to detect S1-dependent shortening of replication tracks in FEN1i-treated cells by DNA spreading, but readily detected such shortening by DNA combing (Fig. 5, A and B).

In conclusion, our data demonstrate that the DNA spreading technique does not resolve the two sister chromatids, whereas DNA combing does. Addressing this question is crucial to properly interpret DNA fiber experiments and to determine whether defects in DNA replication fork progression and/or the maturation of nascent DNA occur in only the leading or lagging strand, or both (Tirman et al., 2021; Quinet et al., 2020; Cong et al., 2021; Bai et al., 2020; Mann et al., 2022; Schrempf et al., 2022; Belan et al., 2022; Simoneau et al. 2021). For example, only the use of DNA combing allows the detection of DNA single-strand gaps and/or DNA double-strand breaks arising in one of two adjacent sister chromatids, resulting from unprocessed Okazaki fragments, DNA replication fork bypass of a template strand DNA lesion, or DNA replication fork collision with a DNA single-strand break.

## Materials and methods

### DNA combing

In the Vindigni lab, DNA combing was conducted as follows. Exponentially growing RPE-1 (human retinal pigment epithelial) cells (RRID:CVCL_4388) were pulse-labeled with CldU (5-Chloro-2′-deoxyuridine; Millipore Sigma) and IdU (5-Iodo-2′-deoxyuridine; Millipore Sigma) as described in the text. Cells were then collected by trypsinization and embedded into agarose plugs, and DNA combing was performed according to the manufacturer’s protocol (Genomic Vision) with minor modifications using the Genomic Vision FiberPrep kit and a Genomic Vision combing machine. The DNA was then baked for 2 h at 60 °C and stored at −20 °C. For immunostaining, DNA was denatured with fresh 0.5 M NaOH and 1 M NaCl solution for 8 min at RT. Coverslips were then washed three times with PBS, dehydrated with 70, 90, and 100% ethanol for 2 min each, and blocked with 10% goat serum in PBS-0.1% Tween-20 at RT for 1 h. DNA fibers were immunostained with rat anti-BrdU for CldU detection (1/75, Ab6326, RRID:AB_305426; Abcam) and mouse-anti-BrdU for IdU detection (1/20, 347580, RRID: AB_400326; BD Biosciences) for 1 h at 37 °C, washed three times with PBS-0.01% Tween-20, and then incubated with anti-rat Alexa Fluor 488 and anti-mouse Alexa Fluor 568 (1/100, A-21470, RRID:AB_2535873 and A-21124, RRID:AB_2535766; Thermo Fisher Scientific, respectively) for 45 min at RT. After three washes with PBS-0.01% Tween-20, coverslips were mounted with Prolong Gold Antifade Reagent (P36930; Thermo Fisher Scientific). For total DNA staining, coverslips were incubated with anti-ssDNA (1/100, MAB3034, RRID:AB_11212688; Millipore) for 1 h at 37 °C, washed, and incubated with anti-mouse Alexa Fluor 647 (1/100, A28181, RRID:AB_2536165; Thermo Fisher Scientific) for 45 min at RT, washed, and then mounted. Images were acquired with LAS AF software using a Leica DMi8 confocal microscope with 40×/1.15 oil immersion objective. The DNA combing assay with the ssDNA-specific S1 nuclease was performed exactly as described above, with the addition of the S1 digestion step as follows. Immediately before combing, the DNA solution was mixed 1:1 with 2× S1 buffer (60 mM sodium acetate pH 4.6, 20 mM zinc acetate, 10% glycerol, and 100 mM NaCl in water) in the presence or absence of 40 U/ml of the S1 nuclease (18001-016; Thermo Fisher Scientific), and incubated at RT for 30 min before combing. Each experiment was performed in duplicate or triplicate as indicated in the figure legend. For each biological replicate, images were taken across the whole coverslip and at least 100–200 fibers were counted using the “multi-point” tool of ImageJ (NIH, https://imagej.nih.gov/ij/, RRID:SCR_018743). Numbers are presented as a percentage of the total count of scored fibers. As a general practice when analyzing fibers, we also look at single channels separately by using the “color balance” function of ImageJ, which allows turning on and off the selected colors. When looking at the single channels, the exposure time is adjusted to account for the different intensities of the IdU and CldU signals. In all cases, when scoring red–green–red or red–green fibers, the red signal is continuous and extends through the green tracts, confirming that our labeling originates from two separate replication cycles. The reason why we do not see yellow tracts when the CldU and IdU overlap is that the IdU signal (green) is stronger than the CldU signal (red) due to the higher efficiency of the antibody used for IdU detection versus the antibody used for CldU detection.

DNA combing in the Caldecott lab was conducted using exponentially growing hTERT-RPE-1 (RRID:CVCL_4388) cells pulse-labeled with 1 μM CldU (5-chloro-2′-deoxyuridine, C6891; Merck) and 250 μM IdU (5-Iodo-2′-deoxyuridine, I7125; Merck) as described in the text (Vaitsiankova et al., 2022). Cells were then collected by trypsinization and resuspended in ice-cold PBS to give a final concentration of 5 × 10^6^ cells/ml. Of this cell mix, 50 μl was prewarmed to 50°C and embedded into an agarose plug (#1703713; BioRad). The plugs were incubated overnight at 42°C in proteinase K lysis buffer (2 mg/ml proteinase K, 10 mM Tris-HCl pH 7.5, 100 mM EDTA, 0.5% SDS, and 20 mM NaCl). Next, the DNA plugs were washed two times for 1 h in TE50 buffer (10 mM Tris-HCl pH 7.5, 50 mM EDTA, and 100 mM NaCl) followed by two times for 1 h in TE buffer (10 mM Tris-HCl pH 7.5, 1 mM EDTA, and 100 mM NaCl). The plugs were then melted at 68°C for 20 min in 1 ml MES (35 mM MES hydrate, 150 mM MES sodium salt, and 100 mM NaCl) and cooled to 42°C for 10 min. The samples were then incubated at 42°C overnight with the addition of 3 μl of β-agarase (M0392L; NEB). The DNA mix was then gently poured into combing reservoirs containing 1.2 ml MES and the genomic DNA was combed onto salinized coverslips (CombiCoverslips, COV-002-RUO; Genomic Vision) using a combing machine (FiberComb; Genomic Vision) and baked for 2 h at 68°C. For immunostaining, DNA was denatured with fresh 0.5 M NaOH 1 M NaCl solution for 8 min at RT. Coverslips were then washed three times with PBS, dehydrated with 70%, 90%, and 100% ethanol for 1 min each, and blocked with 1% BSA in PBS-0.1% Tween-20 at RT for 1 h. DNA fibers were immunostained with rat anti-BrdU for CldU detection (1/30, Ab6326, RRID:AB_305426; Abcam) and mouse-anti-BrdU for IdU detection (1/25, 347580, RRID: AB_400326; BD Biosciences) for 1 h at 37°C, washed three times with PBS-0.01% Tween-20, and then incubated with goat anti-rat Alexa Fluor 568 (1/25, A11077, RRID:AB_2534121; Thermo Fisher Scientific) and goat anti-mouse Alexa Fluor 488 (1/25 A11001, RRID:AB_2534069; Thermo Fisher Scientific) for 45 min at 37°C. After three washes with PBS-0.01% Tween-20, coverslips were dehydrated in ethanol before mounting on microscope slides with fluoroshield (F6182; Merck). The slides were imaged at room temperature using an Apotome widefield microscope (Zeiss) with ×40 oil objectives, 2 XFlash 4.0 Lite camera, and Zeiss Colibri 7 LED. Zeiss Zen (blue) was used for image acquisition, and single-channel TIFF images (8-bit) were exported from the microscope. ImageJ64 software (NIH, https://imagej.nih.gov/ij/, RRID:SCR_018743) was used to visualize, merge the single-channel images, and score the labeled replication tracks. The combing experiment was replicated six times, as indicated in the figure legend. For each biological replicate, images were across the whole of the coverslip and at least 100–200 fibers were counted using the multi-point tool of ImageJ. As a general practice when analyzing fibers, we also look at single channels separately by using the color balance function of ImageJ, which allows turning on and off the selected colors. When looking at the single channels, the exposure time is adjusted to account for the different intensities of the IdU and CldU signals. In all cases, when scoring red–green–red or red–green fibers, the red signal is continuous and extends through the green tracts, confirming that our labeling originates from two separate replication cycles. Numbers are presented as a percentage of the total count of scored fibers.

In the Ray Chaudhuri lab, DNA combing was performed according to the manufacturer’s protocol (Genomic Vision) with minor modifications. Exponentially growing hTERT-RPE1 (RRID:CVCL_4388) cells were pulse-labeled with 30 μM CldU (5-Chloro-2′-deoxyuridine, 105478; MP Biomedicals) and 250 μM IdU (5-Iodo-2′-deoxyuridine, I7125; Sigma-Aldrich) as described in the figure legends. Cells were then collected by trypsinization and embedded into agarose plugs. The plugs were incubated in Proteinase K buffer (10 mM Tris pH 7.5, 100 mM EDTA pH 8.0, 1% N-laurylsarcosine, and 1 mg/ml proteinase K) for 36–48 h at 37°C. The proteinase K buffer was changed twice during this time. The plugs were washed several times in TE50 + 100 mM NaCl buffer (10 mM Tris pH 7.5, 50 mM EDTA pH 8.0, and 100 mM NaCl) and stored at 4°C in the dark until DNA extraction. DNA was extracted by washing the plug for 1 h with 1× TE pH 7.5 + 500 mM NaCl buffer, after which the plug was washed three times for 5 min with MES buffer (50 mM MES hydrate, 50 mM MES sodium salt, and 5 M NaCl). The plug-in MES buffer was incubated at 68°C for 30 min after which it was equilibrated at 42 °C for 5 min. 1,2 U β-agarase (M0392; NEB) per ml MES buffer was added to the solution and incubated at 42 °C overnight. The extracted DNA was combed using a combing machine onto silanized coverslips (CombiCoverslips, COV-002-RUO; Genomic Vision). The combing machine was designed and manufactured by the Department of Experimental Medical Instrumentation (EMI), Erasmus MC with eight sample holders and a retraction rate of 300 μm per second. The coverslips were baked for 2 h at 60 °C and stored at −20 °C. For immunostaining, DNA was denatured with fresh 0.5 M NaOH 1 M NaCl solution for 8 min at RT. Coverslips were then washed three times with PBS, dehydrated with 70%, 90%, and 100% ethanol for 2 min each, and blocked with 3% BSA in PBS at RT in a humidified chamber for 30 min. DNA fibers were immunostained with rat anti-BrdU for CldU detection (1/75, Ab6326, RRID:AB_305426; Abcam) and mouse-anti-BrdU for IdU detection (1/20, 347580, RRID:AB_400326; BD Biosciences) for 45 min at 37 °C in a humidified chamber, washed three times with PBS-0.01% Tween-20, and then incubated with anti-rat Cy3 (1:250, 712-166-153, RRID:AB_2340669; Jackson Immuno-Reasearch Laboratories, Inc.) and anti-mouse Alexa Fluor 488 (1:250, A11001, RRID:AB_10077726; Invitrogen) for 45 min at 37 °C in a humidified chamber. After three washes with PBS-0.01% Tween-20, coverslips were mounted with Prolong Gold Antifade Reagent (P36930; Thermo Fisher Scientific). For total DNA staining, coverslips are incubated with anti-ssDNA (1/100, AB_10805144, RRID:AB_10805144; DSHB) overnight at 4°C in a humidified chamber, washed, incubated with anti-mouse Alexa Fluor 350 (1/100, A11045, RRID:AB_2534100; Thermo Fisher Scientific) for 45 min at RT in a humidified chamber, washed, and then mounted. Images are acquired by Metafer5 from MetaSystems using the Zeiss Imager.Z2 microscope and CoolCube 4 camera at 40×/0.75 (420360-9900). Neon Software from MetaSystems (RRID:SCR_016306) was used for image acquisition, and single-channel TIFF images (8-bit) were exported from the microscope. The single-channel images were merged together using the Combing-RGB macro (https://github.com/CarmenFonseca95/SisterChromatid-JCB) in ImageJ software64. Data analysis was carried out with ImageJ software64 (NIH, https://imagej.nih.gov/ij/, RRID:SCR_018743). Each experiment was performed in triplicate as indicated in the figure legends. For each biological replicate, at least 150 images were taken and 100–200 fibers were counted. As a general practice when analyzing fibers, we also look at single channels separately by using the color balance function of ImageJ, which allows turning on and off the selected colors. When looking at the single channels, the exposure time is adjusted to account for the different intensities of the IdU and CldU signals. In all cases, when scoring red–green–red or red–green fibers, the red signal is continuous and extends through the green tracts, confirming that our labeling originates from two separate replication cycles. Numbers are presented as a percentage of the total count of scored fibers.

### DNA spreading

The DNA fiber spreading assay was performed in the Vindigni lab using exponentially growing RPE-1 (human retinal pigment epithelial) cells (RRID:CVCL_4388) pulse-labeled with CldU (5-Chloro-2′-deoxyuridine; Millipore Sigma) and IdU (5-Iodo-2′-deoxyuridine; Millipore Sigma), as described in the figure legends (Meroni et al., 2022). Cells were then washed twice with PBS, collected by trypsinization, resuspended in PBS for a final concentration of 1,500 cells/μl, and spotted onto positively charged glass slides. Cells were mixed with lysis buffer (200 mM Tris-HCl pH 7.5, 50 mM EDTA, and 0.5% SDS in water), incubated for 5 min at RT, and the slides were tilted at a 20–45° angle to spread the fibers at a constant, low speed. After 10 min air drying, DNA was fixed with a freshly prepared solution of methanol and glacial acetic acid at 3:1 for 5 min. For immunostaining, DNA was rehydrated in PBS twice for 5 min, then denatured with 2.5 M HCl for 1 h at RT. Slides were then washed with PBS three times and blocked with 5% BSA at 37°C for 45 min. For CldU and IdU detection, DNA fibers were immunostained with rat anti-BrdU (1/75, Ab6326, RRID: AB_305426; Abcam) and mouse-anti-BrdU (1/20, 347580, RRID: AB_400326; BD Biosciences) for 1.5 h at RT, respectively. For IdU and total DNA detection, DNA fibers were sequentially immunostained with mouse-anti-BrdU (1/20, 347580, RRID: AB_400326; BD Biosciences) and anti-ssDNA (1/100, MAB3034, RRID:AB_11212688; Millipore) for 1 h at RT, respectively. The slides were then washed three times with PBS-0.05% Tween-20 for 5 min and then incubated with anti-rat Alexa Fluor 488, anti-mouse Alexa Fluor 568, and anti-mouse Alexa Fluor 647 (1/100, A-21470, RRID:AB_2535873, A-21124, RRID:AB_2535766, and RRID:AB_2536165; Thermo Fisher Scientific, respectively) for 1 h at RT. After three washes with PBS-0.05% Tween-20 of 5 min each, slides were mounted with Prolong Gold Antifade Reagent (P36930; Thermo Fisher Scientific). Images were acquired with LAS AF software using a Leica DMi8 confocal microscope with 40×/1.15 oil immersion objective. For the DNA fiber spreading assay with the ssDNA-specific S1 nuclease, after thymidine analogs incorporation, cells were permeabilized with CSK100 (100 mM NaCl, 10 mM MOPS pH 7, 3 mM MgCl_2_, 300 mM sucrose, and 0.5% Triton X-100 in water), treated with the S1 nuclease (18001-016; Thermo Fisher Scientific) at 20 U/ml in S1 buffer (30 mM sodium acetate pH 4.6, 10 mM zinc acetate, 5% glycerol, 50 mM NaCl in water) for 30 min at 37°C, and collected in PBS-0.1%BSA with cell scraper (Meroni et al., 2022). Nuclei were then pelleted at ∼4,600 × *g* for 5 min at 4°C, resuspended in PBS, and processed as intact cells in the standard DNA spreading assay. Each experiment was performed in duplicate or triplicate as indicated in the figure legend. For each biological replicate, images were taken across the whole slide, and at least 100–200 fibers were counted using the multi-point tool of ImageJ (NIH, https://imagej.nih.gov/ij/, RRID:SCR_018743). As a general practice when analyzing fibers, we also look at single channels separately by using the color balance function of ImageJ, which allows turning on and off the selected colors. When looking at the single channels, the exposure time is adjusted to account for the different intensities of the IdU and CldU signals. In all cases, when scoring red–green–red or red–green fibers, the red signal is continuous and extends through the green tracts, confirming that our labeling originates from two separate replication cycles. Numbers are presented as a percentage of the total count of scored fibers.

The DNA spreading assay in the Ray Chaudhuri lab was conducted as follows. Exponentially growing RPE-1 cells (RRID:CVCL_4388) were pulse-labeled with 30 μM CldU and 250 μM IdU as described in the figure legends. Cells were then washed twice with PBS, collected for trypsinization, and resuspended in PBS for a final concentration of 3.5 × 10^5^ cells/ml. Cells were mixed with a drop of lysis buffer on a positively charged glass slide and incubated at RT for 8 min. Slides were then tilted at a 20–45° angle to spread the fibers at a constant, low speed over the slide. After air drying, DNA was fixed with a freshly prepared solution of methanol and glacial acetic acid at a ratio of 3:1 overnight. For immunostaining, DNA was rehydrated in PBS twice for 5 min and then denatured with 2.5 M HCl for 1 h at RT. Slides were then washed with PBS five times and blocked with 5% BSA at RT for 45 min. DNA fibers were immunostained with mouse-anti-BrdU (1/100, 347580, RRID: AB_400326; BD Biosciences) for 1.5 h at RT, washed five times with PBS-0.05% Tween-20, and then incubated with anti-mouse Alexa Fluor 488 (1:250, A11001, RRID:AB_10077726; Invitrogen) for 1 h at RT. After five washes with PBS-0.05% Tween-20, the slides were incubated overnight with anti-ssDNA (1/100, AB_10805144, RRID:AB_10805144; DSHB) at 4°C. The next day the slides were washed, incubated with anti-mouse Alexa Fluor 350 (1/100, A11045, RRID:AB_2534100; Thermo Fisher Scientific) for 45 min at RT in a humidified chamber, washed, and then mounted. Image acquisition was performed as described for the combing assay and the Fiber-GB macro (https://github.com/CarmenFonseca95/SisterChromatid-JCB) was used to merge the single-channel images in ImageJ software64. Each experiment was performed in triplicate as indicated in the figure legends. For each biological replicate, around 150 images were taken and 150–200 fibers were counted. As a general practice when analyzing fibers, we also look at single channels separately by using the color balance function of ImageJ, which allows turning on and off the selected colors. When looking at the single channels, the exposure time is adjusted to account for the different intensities of the IdU and CldU signals. In all cases, when scoring red–green–red or red–green fibers, the red signal is continuous and extends through the green tracts, confirming that our labeling originates from two separate replication cycles. Numbers are presented as a percentage of the total count of scored fibers.

### Hybrid DNA spreading

The hybrid DNA spreading assay in the Ray Chaudhuri lab was conducted as follows. Human RPE-1 cells (RRID:CVCL_4388) were seeded on an 18 × 24 mm coverslip and the next day they were pulse-labeled with 30 μM CldU and 250 μM IdU, as described in the figure legends. Cells were then washed twice before the combing machine was used to lyse and spread the DNA on the coverslips. This was done by attaching the coverslips to the clips of the combing machine and then lowering them into the combing reservoir containing the lysis buffer (200 mM Tris-HCl pH 7.5, 50 mM EDTA, and 0.5% SDS). The coverslips were incubated in the lysis buffer for 5 min after which they were slowly raised to allow the exposed DNA to be spread over the coverslip. After air drying, DNA was fixed with a freshly prepared solution of methanol and glacial acetic acid at the ratio 3:1 overnight. For immunostaining, DNA was rehydrated in PBS twice for 5 min and then denatured with 2.5 M HCl for 1 h at RT. Slides were then washed with PBS five times and blocked with 5% BSA at RT for 45 min. For the CldU/IdU experiment, DNA fibers were immunostained with rat anti-BrdU (1/100, Ab6326, RRID:AB_305426; Abcam) and mouse-anti-BrdU (1/100, 347580, RRID:AB_400326; BD Biosciences) for 1.5 h at RT, respectively, washed five times with PBS-0.05% Tween-2, and then incubated with anti-rat Cy3 (1:250, 712-166-153, RRID:AB_2340669; Jackson Immuno-Reasearch Laboratories, Inc.) and anti-mouse Alexa Fluor 488 (1:250, A11001, RRID:AB_10077726; Invitrogen) for 1 h at RT. After five washes with PBS-0.05% Tween-20, the slides were mounted with Prolong Gold Antifade Reagent. For the IdU/ssDNA experiment, DNA fibers were immune-stained with mouse-anti-BrdU (1/100, 347580, RRID:AB_400326; BD Biosciences) for 1.5 h at RT, washed five times with PBS-0.05% Tween-20, and then incubated with anti-mouse Alexa Fluor 488 (1:250, A11001, RRID:AB_10077726; Invitrogen) for 1 h at RT. After five washes with PBS-0.05% Tween-20, the slides were incubated overnight with anti-ssDNA (1:100, AB_10805144, RRID:AB_10805144; DSHB) at 4°C. The next day the slides were washed, incubated with anti-mouse Alexa Fluor 350 (1:100, A11045, RRID:AB_2534100; Thermo Fisher Scientific) for 45 min at RT in a humidified chamber, washed, and then mounted. Image acquisition was performed just as the combing assay, except that the Fiber-RG or Fiber-RB macro (https://github.com/CarmenFonseca95/SisterChromatid-JCB) was used to merge the single-channel images in ImageJ software64. Each experiment was performed in triplicate as indicated in the figure legends. For each biological replicate, at least 150 images were taken and 100–200 fibers were counted. As a general practice when analyzing fibers, we also looked at single channels separately by using the color balance function of ImageJ, which allows turning on and off the selected colors. When looking at the single channels, the exposure time is adjusted to account for the different intensities of the IdU and CldU signals. In all cases, when scoring red–green–red or red–green fibers, the red signal is continuous and extends through the green tracts, confirming that our labeling originates from two separate replication cycles. Numbers are presented as a percentage of the total count of scored fibers.

### Statistical analysis

Statistical analysis was performed using Prism 8 (GraphPad Software). Details of the individual statistical tests are also indicated in the figure legends and results. In all cases: ns, non-significant; *P < 0.05, ****P < 0.0001. All experiments were repeated for at least three independent biological repeats, as indicated in the figure legends. Statistical differences in the DNA fiber tract lengths were determined by Kruskal–Wallis followed by Dunn’s multiple comparisons test.

### Online supplemental material

Fig. S1 (related to Fig. 2) shows the examples of DNA fiber images collected with the combing, spreading, and hybrid spreading techniques. Fig. S2 (related to Fig. 2) shows the DNA combing and spreading experiments in U2OS cells. Fig. S3 (related to Fig. 4) shows examples of DNA fiber images of the total DNA staining experiments performed by the DNA combing, spreading, and hybrid spreading techniques. Fig. S4 (related to Fig. 4) shows that the addition of proteinase K increases the percentages of broken forks detected by spreading.

## Data Availability

All data described in this study are available in the paper and the supplemental material. Further information and requests for resources and reagents should be directed to A. Ray Chaudhuri (a.raychaudhuri@erasmusmc.nl), K.W. Caldecott (k.w.caldecott@sussex.ac.uk), and A. Vindigni (avindigni@ wustl.edu).
